# Investigation on factors associated with ovarian cancer: an umbrella review of systematic review and meta-analyses

**DOI:** 10.1186/s13048-021-00911-z

**Published:** 2021-11-11

**Authors:** Kiarash Tanha, Azadeh Mottaghi, Marzieh Nojomi, Marzieh Moradi, Rezvan Rajabzadeh, Samaneh Lotfi, Leila Janani

**Affiliations:** 1 Department of Biostatistics, School of Public Health, Iran University of Medical Sciences, Tehran, Iran; 2 Research Center for Prevention of Cardiovascular Diseases, Institute of Endocrinology and Metabolism, Iran University of Medical Sciences, Tehran, Iran; 3 Preventive Medicine and Public Health Research Center, Psychosocial Health Research Institute, Community and Family Medicine Department, School of Medicine,Iran University of Medical Sciences, Tehran, Iran; 4 Department of Sociology & Anthropology, Nipissing University, Ontario North Bay, Canada; 5 Department of Epidemiology, School of Public Health, Iran University of Medical Sciences, Tehran, Iran; 6 School of Health, North Khorasan University of Medical Sciences, Bojnurd, Iran; 7 Imperial Clinical Trials Unit, School of Public Health, Faculty of Medicine, Imperial College London, London, UK

**Keywords:** Ovarian cancer, Risk factor, Protective factor, Nutritional factors, Genetic factors, Environmental factors

## Abstract

Following cervical and uterine cancer, ovarian cancer (OC) has the third rank in gynecologic cancers. It often remains non-diagnosed until it spreads throughout the pelvis and abdomen. Identification of the most effective risk factors can help take prevention measures concerning OC. Therefore, the presented review aims to summarize the available studies on OC risk factors. A comprehensive systematic literature search was performed to identify all published systematic reviews and meta-analysis on associated factors with ovarian cancer. Web of Science, Cochrane Library databases, and Google Scholar were searched up to 17th January 2020. This study was performed according to Smith et al. methodology for conducting a systematic review of systematic reviews. Twenty-eight thousand sixty-two papers were initially retrieved from the electronic databases, among which 20,104 studies were screened. Two hundred seventy-seven articles met our inclusion criteria, 226 of which included in the meta-analysis. Most commonly reported genetic factors were MTHFR C677T (OR=1.077; 95 % CI (1.032, 1.124); P-value<0.001), BSML rs1544410 (OR=1.078; 95 %CI (1.024, 1.153); P-value=0.004), and Fokl rs2228570 (OR=1.123; 95 % CI (1.089, 1.157); P-value<0.001), which were significantly associated with increasing risk of ovarian cancer. Among the other factors, coffee intake (OR=1.106; 95 % CI (1.009, 1.211); P-value=0.030), hormone therapy (RR=1.057; 95 % CI (1.030, 1.400); P-value<0.001), hysterectomy (OR=0.863; 95 % CI (0.745, 0.999); P-value=0.049), and breast feeding (OR=0.719, 95 % CI (0.679, 0.762) and P-value<0.001) were mostly reported in studies. Among nutritional factors, coffee, egg, and fat intake significantly increase the risk of ovarian cancer. Estrogen, estrogen-progesterone, and overall hormone therapies also are related to the higher incidence of ovarian cancer. Some diseases, such as diabetes, endometriosis, and polycystic ovarian syndrome, as well as several genetic polymorphisms, cause a significant increase in ovarian cancer occurrence. Moreover, other factors, for instance, obesity, overweight, smoking, and perineal talc use, significantly increase the risk of ovarian cancer.

**Supplementary Information:**

The online version contains supplementary material available at 10.1186/s13048-021-00911-z.

## Background

Following cervical and uterine cancer, ovarian cancer (OC) has the third rank in gynecologic cancers. A woman’s risk of getting ovarian cancer during her lifetime is about 1 in 78. Mortality rate of ovarian cancer is about 1 in 108. (These statistics don’t count low malignant potential ovarian tumors.) It often remains non-diagnosed until it spreads throughout the pelvis and abdomen, making its treatment even more difficult. At its early stages, when it is limited to the ovary, the treatment success has a higher rate. The silent tumor growth in OC increases its mortality rate and deteriorates its prognosis [1]. OC has a 46 % five-year survival rate. Early detection is important. Most women with Stage 1 ovarian cancer have an excellent prognosis. Stage 1 patients with grade 1 tumors have a 5-year survival of over 90 %, as do patients in stages 1 A and 1B [2].

Besides the undetectable progress of this type of cancer, improper screening methods further delay its diagnosis [3]. Due to the low prevalence of ovarian cancer even amongst postmenopausal women (1:2500), an efficient screening tool requires high sensitivity (>75 %) and extremely high specificity (99.7 %) [4].

A significant increase is estimated in its mortality rate by 2040. Nonetheless, identification of the most effective risk factors can be helpful in prevention measures concerning OC [5]. Conflicting results can be found in the literature describing the role of several factors (e.g., nutritional, environmental, and genetic factors, as well as lifestyle, drug use, and medical history). Genetic predisposition is related to a higher risk of ovarian cancer that also tends to occur at a younger age. BRCA1 and 2 mutation carriers harbor significantly increased ovarian cancer risk (40–45 % resp. 15–20 %) by the age of 70. Risk of OC in the high risk women under 40 years old is low [6]. Several studies on ovarian cancer have been published that have examined various factors influencing the incidence, prevalence and mortality rate. Some of these studies were purely observational and some were meta-analyzes. So far, no study has been published that has summarized and re-analyzed the results of various meta-analyzes in this field, and this issue shows the importance of this study. The present study examined up to 50 factors (nutritional and genetic factors, drugs use, some diseases, breast feeding, smoking and physical activity) that other studies had examined and sometimes presented conflicting results.

The presented umbrella meta-analysis and systematic review is focused on any kind of risk factors on ovarian cancer among all women and aimed to summarize the available reviews and find the most important OC risk factors.

This study is focused on any kind of risk factors on ovarian cancer among all women.

## Methods

A systematic review of systematic reviews was conducted to identify the associated factors with OC. This study was performed according to Smith et al. methodology for conducting a systematic review of systematic reviews [7].

### Study question

What are the most important factors associated with ovarian cancer found in systematic reviews?

### Literature search

A comprehensive systematic literature search was performed to identify all published systematic reviews and meta-analysis on associated factors with OC. Medline through PubMed, Scopus, Embase, Web of Science, Cochrane Library databases, and Google Scholar all were searched up to 17th January 2020 without time limitation. The search strategy included the use of Mesh terms and keywords related to subject and study design (ovarian; ovary; cancer; carcinoma; neoplasm; tumor; Malignancy; review; systematic review; systematic literature review; meta-analysis). The detailed search strategy for the Medline can be found in the supplementary, Table 1 S. The reference lists of selected articles were also manually searched to identify any additional related documents.

### Study selection

This overview only included systematic reviews of factors associated with OC.

The articles which met the following criteria were included in our study: (1) systematic reviews or meta-analysis; (2) have evaluated risk factors of Ovarian cancer; (3) have at least abstracts in English. The articles that were narrative reviews or had assessed prognostic factors of OC or did not provide at least abstract in English were excluded. Characteristic of included studies are illustrate in Table 1.

**Table 1:** Characteristic of included studies

No.	Author	Year	No. of Articles	No. of Patient (total)	No. of Cases	No. of Control	Evaluated Factors
1	Yan Qiao	2018	21	309	-	-	Aspirin
2	Hongmei Chen	2017	14	11,690	4448	7242	VDR rs2228570
3	Li-Hui Yan	2018	46	84,772	36,298	48,474	BRCA2 N372H
4	Jie Ruan	2018	24	1217	-	-	P16INK4a
5	liang Tang	2018	13	13,064	5461	7603	HER2 and ESR2 polymorphisms
6	Ross Penninkilampi	2018	27	-	14,311	-	Talc Use
7	Chao-Huan Xu	2017	7	3016	1,345	1,671	Genetic polymorphisms
8	Xu-Ming Zhu	2017	10	4621	1930	2464	Genetic polymorphisms
9	JieNa Li	2017	9	4024	1333	2691	ERCC2 rs13181
10	Jing Li	2017	7	-	1898	-	C-reactive protein
11	Dongyu Zhang	2017	14	2,342,245	4184	‬‬	Diabetes mellitus
12	Xingxing Song	2017	15	493,415	7453	485,962	Calcium Intake
13	Wera Berge	2016	27	34,176	15,154	19,022	Talc Use
14	Xin Zhan	2017	18	701,857	8,683	693,174	Tea consumption
15	A Darelius	2017	11	-	-	-	Hysterectomy
16	Zhiyi Zhou	2017	13	2,951,539	13,616	2,937,923	Pelvic inflammatory disease
17	Yang Deng	2017	8	14,014	6613	7401	Androgen receptor gene
18	Bamia Christina	2016	32	-	11,411	-	Coffee Intake
19	Lihua Wang	2017	13	3,708,313	5534	3,702,779	Diabetes mellitus
20	lilin he	2017	8	45,624	19,260	26,364	MTHFR C677T
21	Chunpeng Wang	2016	38	409,061	40,609	368,452	Endometriosis, Tubal Ligation, Hysterectomy
22	Chunyan Shen	2016	12	1235	806	429	Adenomatous polyposis coli (APC) gene
23	Xiyue Xiao	2016	12	901	612	289	P16INK4a
24	Fangfang Zeng	2016	7	33,456	2011	31,445	Inflammatory markers
25	Dongyu Zhang	2016	23	499,950	15,163	484,787	Aspirin
26	Wenlong Qiu	2016	25	900,000	6612	893,388	Dietary fat intake
27	Qiang Wang	2016	9	740	485	255	CDH1 promoter
28	Xiaoli Hua	2016	12	2,361,494	6,275	2,355,219	Dietary Flavonoids
29	Li-feng Shi	2015	12	2,353,945	8896	2,345,049	Hormone therapy
30	Christos Iavazzo	2016	4	725	385	340	Hypodontia
31	Sang-Hee Yoon	2016	3	5,659,211	3509	5,655,702	salpingectomy
32	Wei Liu	2016	35	42,650	19,527	23,123	A1298C POLYMORPHISM
33	Vida Mohammadi	2019	7	381,810	3653	378,157	flavonoids
34	Lifeng Li	2016	9	-	-	-	Metformin
35	Arefe Parvaresh	2019	13	-	-	-	Quercetin
36	Xiaowei Yu	2016	14	11,471	3796	7675	ERCC2 rs13181 - XRCC2 rs3218536
37	Rui Hou	2015	20	1,117,992	12,046	1,105,946	Dietary fat
38	Zhen Liu	2015	26	34,817	12,963	21 854	overweight, obesity
39	N. Keum	2015	18	-	2636	-	Egg intake
40	Liangxiang Su	2015	4	12,016	2344	9672	BRCA2 N372H
41	Sai-tian Zeng	2014	12	629,453	3728	625,725	Egg intake
42	Xiaolian Zhang	2015	5	4233	1791	2,196	Vascular Endothelial Polymorphisms
43	Li-Ping Feng	2014	19	469,095	9438	459,657	Breastfeeding
44	collaborative Group	2015	52	12,110	-	-	Menopausal hormone use
45	Huang Yan-Hong	2015	13	1,996,841	5857	1,990,984	alcohol consumption
46	Jiyi Hu	2015	8	305,338	3555	301,783	cruciferous vegetables
47	Jing Liao	2014	21	3117	2842	4305	progesterone receptor Polymorphisms
48	Xingzhong Hu	2015	5	5884	2336	3548	RAD51 Gene 135G/C
49	Jing Liu	2014	19	-	-	-	Milk, Yogurt, and Lactose Intake
50	Jun Qin	2014	62	92,857	42,315	50,542	STK15 polymorphisms
51	Luliang Liu	2015	15	14,798	7,450	7,348	MMP-12-82 A/G polymorphism
52	X.Y. Shi	2015	3	7026	-	-	MTHFR A1298C polymorphism
53	M. Zhai	2015	4	10,169	3565	6604	Arg188His polymorphism
54	Yue-Dong Wang	2014	15	1653	822	831	serum levels of osteopontin
55	John A. Barry	2014	3	72,973	919	72,054	polycystic ovary syndrome
56	Xinli Li	2014	10	72,054	6127	65,927	dietary lycopene intake
57	Xue Qin	2014	4	1133	474	659	Asn680Ser polymorphism
58	Shujing Shi	2014	13	16,230	5,927	10,303	RAD51 135 G>C and XRCC2 G>A (rs3218536)
59	M. A. Alqumber	2014	12	2257	993	1264	72 Arg.Pro Polymorphism
60	Pei-yue Jiang	2014	15	889,033	6,087	882,946	Fish Intake
61	Danhua Pu	2014	7	7356	3493	3863	MTHFR Polymorphism
62	Xinwei Pan	2013	8	7724	3,723	4,001	Ala222Val
63	Yulan Yan	2013	4	9108	3,635	5,473	XRCC3 Thr241Met polymorphism
64	Tracy E. Crane	2013	24	519,431	2091	517,340	Dietary Intake
65	Su Li	2014	14	10,964	-	-	VDR rs2228570
66	Dan Cheng	2014	22	15,343	6836	8507	RAD51 Gene 135G/C polymorphism
67	Bo Han	2014	11	379,868	4,306	375,562	Cruciferous vegetables
68	Xin-Lan Qu	2014	10	297,892	4392	293,500	Phytoestrogen Intake
69	Jin-Ze Du	2014	8	3940	1,293	2,647	COMT rs4680 Polymorphism
70	Li-Yuan Han	2014	10	6001	2578	3423	GST Genetic Polymorphisms
71	Da-Peng Li	2014	40	415,949	17,139	398,810	Breastfeeding
72	Yong-Jun Ma	2014	6	3839	1,766	2,073	Rs11615 (C>T)
73	Jalal Poorolajal	2014	19	-	-	-	BMI
74	Li-Min Zhou	2014	6	435,398	2983	432,415	Recreational Physical Activity
75	Piyemeth Dilokthornsakul	2013	4	-	-	-	Metformin
76	Chenglin Li	2013	18	227,859	5677	222,182	Folate intake and MTHFR polymorphism C677T
77	Susan J. Jordan	2013	22	-	-	-	hysterectomy
78	Nan-Nan Luan	2013	35	720,617	14,465	706,152	Breastfeeding
79	Xue Qin	2013	7	4,809	1977	2832	VDR
80	Laura J. Havrilesky	2013	55	31,056	10,031	21,025	Oral Contraceptive
81	Ting-Ting Gong	2012	27	1,020,516	9859	1,010,657	Age at menarche
82	Yanling Liu	2013	6	10,768	4,107	6,661	VDR
83	Louise Baandrup	2012	21	563,976	11,759	552,217	NSAIDs
84	Jung-Yun Lee	2012	19	-	-	-	Diabetes Mellitus
85	Chengbin Ma	2013	10	18, 628	5, 932	12,696	MTHFR C677T polymorphism
86	Ying-Yu Ma	2013	6	3745	1534	2211	MDM2 309T.G Polymorphism
87	Gwan Gyu Song	2013	12	8775	3716	5059	VDR
88	Ketan Gajjar	2012	5	3795	1199	2596	Cytochrome P1B1 (CYP1B1)
89	Xiaojian Ni	2012	17	193,424	10 373	183,051	NSAIDs
90	Lu Liu	2012	4	7127	3,496	3,631	C677T and A1298C polymorphism
91	T.N. Sergentanis	2012	11	5025	1,680	3345	MspI and Ile462-Val and Thr461Asn
92	*Collaborative Group*	2012	51	123,056	28 114	94,942	Smoking
93	Megan S Rice	2012	30	18,929	-	-	Tubal ligation and Hysterectomy
94	Matteo Rota	2012	27	15,762,134	16,554	15,745,580	Alcohol drinking
95	Collaborative Group	2012	47	106,468	25,157	81,313	Body Size
96	Su-Qin Shen	2012	18	7368	2,193	5,175	TP53 Arg72Pro
97	Xiao-Ping Ding	2012	8	7457	3,379	4,078	MTHFR C677T Polymorphism
98	M.G.M. Braem	2011	150	-	-	-	Genetic variants
99	*M. Constanza Camargo*	2011	18	21,973	117	22,090	Asbestos
100	David Cibula	2011	3	-	-	-	Oral contraceptives
101	Sarah J. Oppeneer	2011	16	-	7234	-	Tea Consumption
102	Lu Yin	2011	10	157,292	-	-	Circulating vitamin D
103	A Wallin	2011	8	754 836	2349	752,487	Red and processed meat consumption
104	D. Cibula	2011	13	-	-	-	Tubal ligation
105	Ru-Yan Liao	2010	4	15,104	5532	9572	TGFBR1*6A/9A polymorphism
106	Linda S. Cook	2010	20	-	-	-	vitamin D
107	K. P. Economopoulos (2010)	2010	2	4240	2049	2191	Meat, fish
108	Hee Seung Kim	2010	10	135,871	65,578	70,293	Wine
109	S-K Myung	2009	7	169 051	3516	165 535	Soy intake
110	BG Chittenden	2009	1	4547	476	4071	Polycystic ovary syndrome
111	Bo Zhou	2008	27	1,584,610	12,955	1,571,655	Hormone replacement therapy
112	HG Mulholland	2008	2	-	-	-	Dietary glycemic index
113	Catherine M. Olsen	2007	12	2778	1269	1509	Recreational Physical Activity
114	J Steevens	2007	21	-	280	-	Tea and coffee drinking
115	C. M. Greiser	2007	42	48,153	12 238	‬‬	Menopausal hormone therapy
116	Catherine M. Olsen	2007	28	1,640,615	53,182	1,587,433	Obesity
117	S. J. Jordan	2006	9	6474	910	5564	smoking
118	Stefanos Bonovas	2005	8	746,293	‬‬	741,888	Paracetamol
119	Susanna C. Larsson	2006	21	-	-	-	Milk, milk products and lactose intake
120	Grimes DA	2009	3	500	-	-	Oral contraceptives
121	Stefanos Bonovas	2005	10	320,544	3803	316,741	Nonsteroidal anti-inflammatory drugs
122	L-Q Qin	2005	22	134,406	8372	126,034	Milk/dairy products consumption
123	Sonya Kashyap	2004	10	13,480	3624	9856	Assisted Reproductive Technology
124	M. Huncharek	2003	16	11,933	-	-	Cosmetic talc
125	V Bagnardi	2001	235	117 471	235	‬‬	Alcohol drinking
126	Michael Huncharek	2009	8	6,689	2529	4160	Dietary Fat Intake
127	S. S. Coughlin	2000	15	-	-	-	Estrogen replacement therapy
128	Pushkal P. Garg	1998	9	259,794	4392	255,402	Hormone replacement therapy
129	John F. Stratton	1998	15	-	6077	-	Family history
130	Bowen Zheng	2018	13	142,189	5777	136,412	Dietary fiber intake
131	Hai-Fang Wang	2017	22	1,485,988	-	-	Empirically derived dietary patterns
132	Hui Xu	2018	19	567,742	-	-	Dietary fiber intake
133	Dongyu Zhang	2018	14	180,833	7500	‬‬	Non-herbal tea consumption
134	Yun-Long Huo	2018	6	81,791	7878	73,913	antidepressant medication
135	Massimiliano Berretta	2018	9	787,076	3,541	‬‬	Coffee consumption
136	Jiaqi Li	2018	7	65,754	-	-	vitamin D receptor
137	Xianling Zeng	2018	11	9987	4097	5890	RAD51 135 G/C polymorphism
138	Marieke GM Braem	2012	3	330,849	1244	329,605	Coffee and tea consumption
139	Shanliang Zhong	2014	19	730,703	9,459	‬‬	Nonoccupational physical activity
140	Xiumin Huang	2018	17	149,177	7609	73,168	dietary fiber intake
141	Ting Liu	2013	17	16,363	6,365	9,998	Progesterone receptor PROGINS
142	Yanyang Pang	2018	10	2354	-	-	Dietary protein intake
143	Ke Wei Foong	2017	43	3,491,943	-	-	Obesity
144	Lingling Zhou	2015	2	774	389	385	SNP rs763110
145	Rizzuto I	2013	25	182,972	-	-	ovarian stimulating drugs for infertility
146	Yanqiong Liu	2014	5	624	-	-	Statin
147	Ahmad Sayasneh	2011	8	-	653	-	Endometriosis
148	Jia li	2018	25	957,152	-	-	Endometriosis
149	Ho Kyung Sung	2016	32	530,950	7639	523,311	Breastfeeding
150	Mahdieh Kamali	2017	17	10,817	4464	6353	XRCC2 rs3218536
151	Menelaos Zafrakas	2014	16	-	17,445	-	Endometriosis
152	Dagfinn Aune	2015	28	-	-	-	Anthropometric factors
153	QIAO WANG	2015	4	1985	627	1358	circulating insulin
154	Yihua Yin	2013	11	6192	2,673	3519	glutathione S-transferase
155	Ximena Gianuzzi	2016	14	8130	1,149	6981	Insulin growth factor (IGF)
156	Li-Ling Liu	2014	4	2675	1073	1602	transforming growth factor b receptor
157	Yong-qiang Wang	2012	4	580,581	2444	578,137	TGFBR1 Polymorphisms
158	Dongyang Li	2018	44	1,082,092	48,345	1,033,747	Dietary inflammatory index
159	Si Huang	2018	10	4605	2394	2211	miR-502-binding site
160	Eileen Deuster	2017	200	-	-	-	VDR
161	Ru Chen	2017	28	3362	2,171	1191	MGMT Promoter
162	Joanna Kruk	2017	26	-	-	-	Dietary alkylresorcinols
163	Xue-Feng Li	2017	11	33,209	14,030	19,179	lncRNA H19 polymorphisms
164	Yan Jiang	2017	1	285	165	120	ARLTS1 polymorphism
165	Qiuyan Li	2017	7	-	-	-	BRCA2 rs144848 polymorphism
166	Mohamed Hosny Osman	2017	1	2,116,029	7124	2,108,905	Cardiac glycosides
167	Erjiang Zhao	2017	4	-	-	-	Glutathione S-transferase
168	Giuseppe Grosso	2017	4	-	-	-	Diet
169	Limin Miao	2017	6	6027	2156	3871	BRCA1 P871L polymorphism
170	Na-Na Yang	2017	4	2110	944	1166	XRCC1 polymorphism
171	Giuseppe Grosso	2016	53	-	-	-	Dietary flavonoid
172	Juan Enrique Schwarze	2017	4	-	-	-	Reproduction technologies
173	Rosanne M. Kho	2016	10	-	-	-	Hysterectomy
174	K Robinson	2016	11	-	-	-	Bisexual
175	Hong-Bae Kim	2016	6	1937	-	-	Benzodiazepine
176	Chuanjie Zhang	2017	3	2628	1276	1352	NFκB1-94ins/del ATTG
177	Minjie Chu	2016	2	18,540	6,857	11,683	H19 lncRNA
178	Duan Wang	2016	4	3036	1463	1573	NFKB1 −94 ins/del ATTG
179	Jun Wang	2016	19	3,87,71,388	13,116	38,758,272	BMI
180	Yun-Feng Zhang	2015	1	549	229	320	IL-27 Genes
181	Ping Wang	2016	2	-	-	-	MDM2 SNP285
182	Wenkai Xia	2015	4	1248	497	751	ESR2
183	Lei Chen	2016	2	-	-	-	L55M polymorphism
184	Davide Serrano	2015	3	5456	2313	3143	VDR
185	Ranadip Chowdhury	2015	41	-	-	-	Breastfeeding
186	Zhi-Ming Dai	2015	3	3530	1475	2055	VDR
187	Claudio Pelucchi	2014	4	-	2,010	-	Dietary acrylamide
188	Yu-Fei Zhang	2015	6	619 714	2933	‬‬	Tea consumption
189	Jin-Lin Cao	2015	2	9245	3102	6143	TERT Genetic Polymorphism
190	Myung-Jin Muna	2015	6	‬‬	4107	6661	VDR
191	NaNa Keum	2015	6	-	-	-	Weight Gain
192	Sheng-Song Chen	2015	2	1185	556	629	MMP-12 82 A/G polymorphism
193	Bei-bei Zhang	2014	45	57,328	28,956	28,372	Genetic 135G/C polymorphism
194	Sara Raimondi	2014	5	97,275	45,218	52,057	BsmI polymorphism
195	Shang Xie	2014	15	11,644	5873	5771	LIG4 gene polymorphisms
196	Wen-Qiong Xue	2014	4	‬‬	36,299	48,483	BRCA2 N372H
197	Patrizia Gnagnarella	2014	6	10,588	4051	6537	VDR
198	Peter Boyle	2014	2	-	-	-	Sweetened carbonated beverage consumption
199	Tara M. Friebel	2014	5	-	-	-	BRCA1 and BRCA2
200	Xin Wang	2014	41	42,121	17,814	24,307	FAS rs2234767G/A Polymorphism
201	Yeqiong Xu	2013	7	11,009	4210	6799	VDR
202	H S Kim	2014	35	444 255	-	-	Endometriosis
203	Yazhou He	2014	7	69,524	30,868	38,656	XRCC2 Arg188His Polymorphismc
204	Weifeng Tang	2014	14	27,269	11,245	16,024	Aurora-A V57I (rs1047972) Polymorphism
205	Yeqiong Xu	2014	3	937	457	480	Polymorphisms
206	Mengmeng Zhao	2014	42	39,505	19,142	20,363	Rad51 G135C
207	Xiao Yang	2014	21	‬‬	6127	9238	NFKB1 −94ins/del ATTG Promoter
208	Bai-Lin Zhang	2014	7	-	9956	-	Blood Groups
209	Ursula Schwab	2014	-	-	-	-	Dietary fat on cardiometabolic
210	Tie-Jun Liang	2013	21	8720	3,498	5,222	137G>C polymorphism
211	Wei Wang	2013	39	41,698	19,068	22,630	RAD51 135 G.C Polymorphism
212	Lei Xu	2013	47	43,295	19,810	23,485	FASL rs763110 Polymorphism
213	Jingxiang Chen	2013	19	48,670	14,814	33,856	TCF7L2 Gene Polymorphism
214	Monica Franciosi	2013	53	1,050,984	-	-	Metformin
215	Zhou Zhong-Xing	2013	41	42,169	17,858	24,311	FAS-1377 G/A (rs2234767) Polymorphism
216	Zhibin Yu	2013	73	38,278	15,942	22,336	Interleukin 10 - 819 C/T Polymorphism
217	Shangqian Wang	2013	2	1706	794	912	PAI-1 4G/5G Polymorphism
218	Li Li Li	2013	8	746,455	-	-	Fertilization
219	XIN XU	2012	21	17,623	8,415	9,208	PAI-1 promoter
220	Dominique Trudel	2012	22	-	-	-	Green tea
221	Tian-Biao Zhou	2012	6	2,658	1,461	1,197	Gene Polymorphism
222	Xin-Min Pan	2011	17	27,759	13 691	14 068	MLH1 -93 G>A polymorphism
223	*Jane Green*	2011	-	-	4830	-	Height
224	C. Pelucchi	2011	3	-	1594	-	Acrylamide
225	Bo Peng	2010	4	1240	443	797	Polymorphisms
226	Bahi Takkouche	2009	10	-	-	-	Hairdressers
227	Bahi Takkouche	2005	2	556	238	318	Hair Dyes
228	V. G. Kaklamani	2003	1	907	659	248	TGFBR1*6A
229	Song Mao	2018	3	-	-	-	klotho expression
230	Mukete Franklin Sona	2018	15	1 915 179	31 893	1,911,045	Type 1 diabetes mellitus
231	Christine Schwarz	2018	4	-	-	-	Night shift work
232	Xiaoqing Shi	2018	-	1208	604	604	NME1 polymorphisms
233	H.J. van der Rhee	2006	2	-	-	-	Sunlight
234	Nadin Younes	2018	44	-	805	-	Polymorphisms
235	Yue Xu	2016	1	-	-	-	BHMT gene rs3733890
236	Zhong Tian	2013	46	51,413	22,993	28,420	CYP1A2*1F polymorphism
237	Yu Wang	2018	1	79,988	-	-	Renal transplants
238	T. O. Yang	2014	-	453 023	2009	451,014	Birth weight
239	Lanhua Tang	2017	-	-	-	-	Night work
240	Steven M. Koehler	2012	8	-	-	-	BMP-2
241	Yan Zhang	2013	9	5632	2,331	3,301	VDR
242	Ivana Rizzuto	2013	25	182,972	-	-	Stimulating drugs for infertility
243	Xiao-san Zhang	2018	7	105,507	6783	98,724	Bisphosphonates use
244	Yun Ye	2018	10	1045	-	-	B7-H4 expression
245	Junga Lee	2018	34	-	-	-	Physical activity
246	Huijun Yang	2019	26	1,174,527	11 410	1 163 117	Age at menarche
247	M. Kadry Taher	2019	27	214,447	15,303	199,144	Perineal use of talc powder
248	Yanjun Wu	2019	13	2,471,030	19,959	2,451,071	Age at last birth
249	A. Moazeni-Roodi	2019	19	37,036	13,562	23,474	MDM2 40 bp indel polymorphism
250	Fateme Shafiei (2018)	2019	22	40 140	8568	31,572	Caffeine
251	Lindsay J. Wheeler	2019	11	13,591	4,484	9,107	Intrauterine Device Use
252	Yuhang Long	2019	16	437,689	4,553	433,136	vitamin C intake
253	M. Arjmand (2020)	2019	16	4184	1106	3078	Circulating omentin levels
254	Claudia Santucci	2019	37	-	70,646	-	smoking
255	A. Salari-Moghaddam	2019	14	-	4434	-	Caffeine
256	M. Karimi-Zarchi	2019	11	12,720	4990	7730	MTHFR 677 C>T Polymorphism
257	Fan Yang	2019	2	445	-	-	ERCC1 gene polymorphisms
258	Tingting Yang	2019	3	-	-	-	Work Stress
259	Youxu Leng	2019	14	-	4597	-	vitamin E
260	Jalal Choupani	2019	4	9532	843	110	mir-196a-2 rs11614913
261	Xiaqin Huo	2019	18	-	14,440	-	Hysterectomy
262	A. Bodurtha Smith	2019	58	292,730	528	292,202	HIV
263	Alireza Sadeghia	2019	21	900,000	-	-	Dietary Fat Intake
264	Kui Zhang	2019	13	40,404	6449	33,955	Fermented dairy foods
265	Zohre Momenimovahed	2019	20	-	-	-	Fertility Drugs
266	Christina Bamia	2019	31	-	13,111	-	Coffee consumption
267	Boris Janssen	2019	115	-	-	-	predicted pathogenic PALB2
268	Yang Liu	2019	12	1,193,201	-	-	Menopausal Hormone Replacement
269	Javaid Iqbal	2018	2	5093	1114	3979	Hormone Levels
270	Sen Li	2019	12	12,933	5057	7876	Genetic polymorphism of MTHFR C677T
271	Guisheng He	2019	45	1,059,975	329,035	730,940	TERT rs10069690 polymorphism
272	Yizi Wang	2019	36	4, 229,061	-	-	Statin use
273	Jun Yu	2019	83	21,612	-	-	SFRP promoter hypermethylation
274	Qiao Wen	2019	7	1,710,080	-	-	Metformin
275	Suszynska M1	2019	5	3748	1919	1829	EPHX1 polymorphism rs1051740
276	Tian Xu1	2019	21	29,981	13,675	16,306	HOTAIR polymorphisms
277	Jinghua Shi	2018	13	901,287	-	-	Metformin

Four authors (RR, MM, SL, and KT) independently screened the titles and abstracts of citations to identify potentially relevant studies. Then, the full texts of potentially eligible articles were obtained and reviewed for further assessment according to the inclusion and exclusion criteria. Controversies were resolved by consulting a third person (LJ).

### Data extraction

Data were extracted from eligible studies using a prespecified form in Microsoft Excel by four authors (RR, MM, SL, and KT) independently. The following information was collected: first author, year of publication, number of included primary studies, number of participants, age of participants, factors associated with OC, besides the measure of association (e.g., RR, OR), and its confidence intervals. Any discrepancy was resolved through discussion with a third author (LJ). EndNote X9 was used to extracting the records and removing duplicates (The EndNote Team. EndNote. EndNote X9 ed. Philadelphia, PA: Clarivate; 2013.).

### Risk of bias assessment

**Fig. 1:**
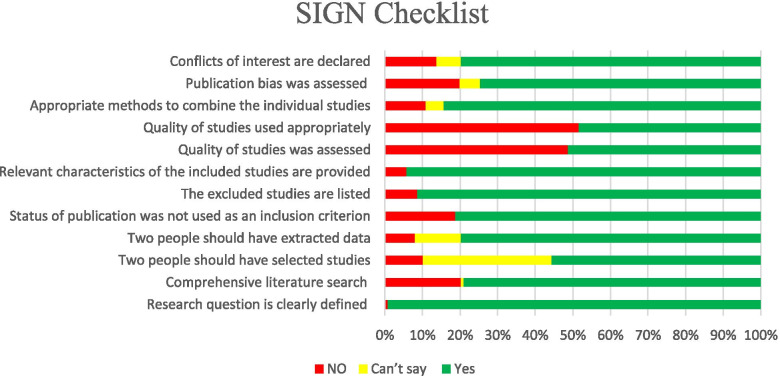
SIGN Checklist scoring

The SIGN checklist was used to assess the methodological quality of systematic reviews (2); it is composed of 12 items containing ‘yes,‘ ‘no,‘ ‘can’t,‘ or ‘not applicable’ options. Generally, the methodological quality of the studies in this checklist was categorized into low quality, acceptable, and high quality, (Fig. 1).

The quality assessment of the eligible studies was undertaken independently by four authors (RR, MM, SL, and KT). Any disagreements were resolved through discussion.

### Data synthesis

All statistical analyses were performed using Stata version 16 (StataCorp. 2019. Stata Statistical Software: Release 16. College Station, TX: StataCorp LLC.).

Most of the studies reported measures of the association between each factor and OC using the odds ratio (OR) or risk ratio (RR) with their corresponding CIs. Only one study used a standardized incidence rate ratio (SIR) and standardized mean difference (SMD) as an effect size. Thus, OR or RR and 95 % confidence intervals (CIs) were used to present the association between the factors and OC. For conducting the meta-analysis, all related information about measures of association (e.g., Pooled OR, Pooled RR, Standard error, 95 % Confidence Interval) were extracted and converted to pooled effect size and its SE for every factor in each study.

Since the reported combined effects from systematic reviews were used in the analysis, so primary studies may have been included in different systematic reviews and meta-analyses in the different years which we were not able to exclude them in the analysis. Heterogeneity was evaluated among the primary studies using the forest plots, Cochran’s Q statistic, and I^2^ statistic. A random-effects model using restricted maximum-likelihood was used if heterogeneity was high (I^2^ > 50 %); otherwise, a fixed-effects model was applied.

Since the number of first reviews combined for the meta-analysis was less than 10, Egger’s regression asymmetry tests were used for assessing the publication bias instead of funnel plots (Egger et al., 1997), where p <0.10 was considered as evidence of bias. The characteristics of the included studies were descriptively summarized using a structured table.

## Results

**Fig. 2:**
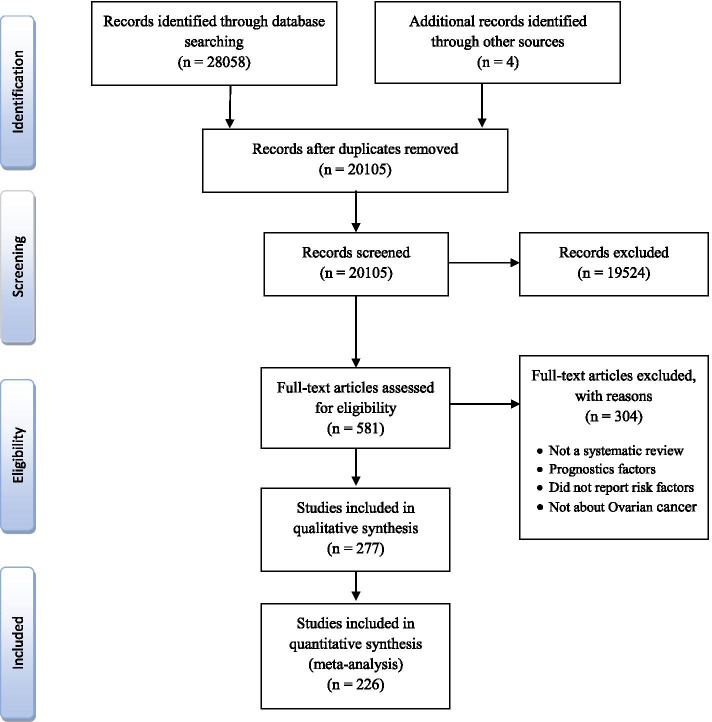
PRISMA flow diagram

Twenty-eight thousand sixty-two papers were initially retrieved from the electronic databases, among which 20,104 studies were screened. Two hundred seventy-seven articles met our inclusion criteria, 226 of which included in the meta-analysis (Fig. 2). The eligible articles were those published between 1998 (when meta-analyses in this field first became available) and 2020. All of the studies had utilized a healthy control group against women with OC.

Overall, from the 277 eligible meta-analyses or systematic reviews, 216 putative risk/protective factors of OC were reported.

Due to the number of evaluated factors, all were categorized into 5 main groups: (1) Nutritional factors, (2) Drug use and Medical history, (3) Diseases, (4) Genetic factors, (5) Other factors.

Among all of the studied factors, 109 had one quantitative synthesis report, and 53 did not have any quantitative synthesis of individual findings but reported valuable data in systematic review articles (Table 2 S and Table 3 S).

### Meta-analysis results of the outcomes of interest

**Fig. 3:**
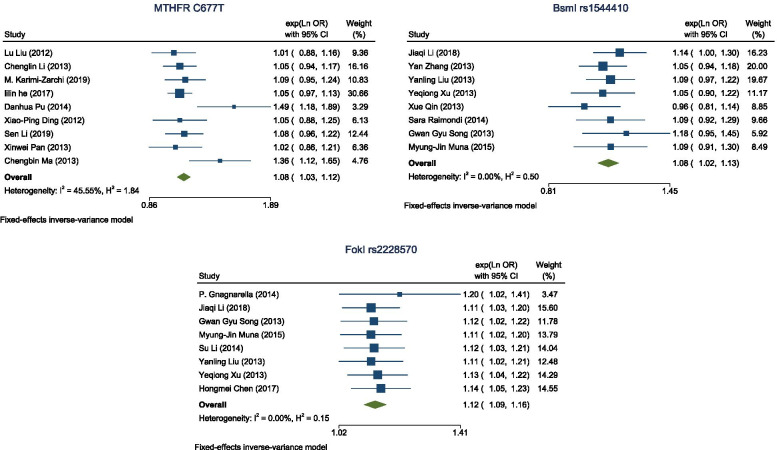
Meta-analysis of OR for MTHFR C677T, BSML rs1544410 and Fokl rs2228570

Meta-analyses were conducted on the 53 associated factors with OC with sufficient data (two or more reports with the same measures). Most commonly reported genetic factors were MTHFR C677T (OR=1.077; 95 % CI (1.032, 1.124); P-value<0.001), BSML rs1544410 (OR=1.078; 95 %CI (1.024, 1.153); and P-value=0.004) and Fokl rs2228570 (OR=1.123; 95 % CI (1.089, 1.157); P-value<0.001), which were significantly associated with increasing risk of OC **(**Fig. 3**).** The results of publication bias assessed using the Egger’s test indicate significant publication bias only for MTHFR C677T factor (P-value=0.017).

Among the other factors, coffee intake (OR=1.106; 95 % CI (1.009, 1.211); P-value=0.030), hormone therapy (RR=1.057; 95 % CI (1.030, 1.400); P-value<0.001), hysterectomy (OR=0.863; 95 % CI (0.745, 0.999); P-value=0.049), and breast feeding (OR=0.719; 95 % CI (0.679, 0.762); P-value<0.001) were mostly reported in studies. Final results of all conducted meta-analysis are presented in Table 2.

**Table 2:** Results of all conducted meta-analysis

Variables	Measure of Association	Odds Ratio (95 % CI)	P-value	I^2^ %	No. of study in analysis	
*Nutritional factors*						
Alcohol use	RR	1.015 (0.974 – 1.052)	0.485	0.01	3	
Coffee intake	OR	1.106 (1.009 – 1.211)	0.030	0.00	4	
	RR	1.036 (0.967 – 1.109)	0.317	0.00	3	
Egg intake	RR	1.147 (1.045 – 1.250)	<0.001	17.73	2	
Fat intake	RR	1.188 (1.090 – 1.296)	<0.001	0.00	3	
Fiber intake	OR	0.760 (0.714 – 0.810)	<0.001	0.00	3	
Milk intake	RR	1.016 (0.664 – 1.554)	0.941	0.08	2	
Tea intake	OR	0.833 (0.741 – 0.936)	0.002	0.00	3	
	RR	0.856 (0.779 – 0.959)	0.005	0.00	2	
Vegetables intake	RR	0.896 (0.837 – 0.958)	<0.001	0.00	2	
*Drug use and Medical history*						
Aspirin	OR	0.894 (0.854 – 0.935)	<0.001	0.00	3	
Metformin	RR	0.718 (0.602 – 0.855)	<0.001	0.00	3	
NSAIDs	RR	0.898 (0.819 – 0.984)	0.020	0.00	3	
Oral contraceptive	OR	0.655 (0.515 – 0.833)	<0.001	78.23	2	
Statin	RR	0.849 (0.749 – 0.962)	0.010	0.00	2	
Hormone therapy (estrogen)	RR	1.305 (1.210 – 1.407)	<0.001	0.00	2	
Hormone therapy (Overall)	RR	1.057 (1.030 – 1.400)	<0.001	94.44	4	
Hormone therapy (estrogen-progestin)	OR	1.190 (1.043 – 1.357)	0.009	82.24	2	
Hysterectomy	OR	0.863 (0.745 – 0.999)	0.049	67.12	4	
Tubal ligation	OR	0.693 (0.657 – 0.731)	<0.001	0.00		
*Diseases*						
Diabetes	RR	1.24 (1.32 – 1.35)	<0.001	0.00	3	
Endometriosis	OR	1.433 (1.294 – 1.586)	<0.001	3.05	2	
Poly cystic ovarian syndrome	OR	1.580 (1.081 – 2.310)	0.018	29.48	2	
*Genetic factors*						
Asn680Ser	OR	1.120 (0.594 – 2.110)	0.726	86.32	2	
BRCA2 N372H rs144848	OR	1.079 (1.018 – 1.143)	0.010	44.61	4	
BSML rs1544410	OR	1.078 (1.024 – 1.153)	0.004	0.00	8	
ESR2 rs3020450	OR	0.818 (0.719 – 1.040)	0.151	61.20	2	
Fokl rs2228570	OR	1.123 (1.089 – 1.157)	<0.001	0.00	8	
GSTM1	OR	1.015 (0.928 – 1.111)	0.741	0.00	2	
MTHFR A1298C	OR	0.997 (0.943 – 1.054)	0.907	0.00	3	
MTHFR C677T	OR	1.077 (1.032 – 1.124)	<0.001	45.55	9	
NFƙB1	OR	1.680 (1.08 – 2.62)	0.020	69.07	2	
P16INK4a	OR	2.657 (1.173 – 6.014)	0.019	51.28	2	
RAD51 135G-C	OR	0.996 (0.922 – 1.075)	0.910	0.00	4	
ERCC1 rs11615	OR	0.987 (0.756 – 1.287)	0.920	0.00	2	
ERCC2 rs13181	OR	1.42 (1.15 – 1.76)	0.001	0.00	2	
VGEGF rs699947	OR	0.983 (0.644 – 1.502)	0.938	78.04	2	
VDR rs731236	OR	0.996 (0.882 – 1.125)	0.842	56.81	6	
FASL rs763110	OR	0.640 (0.520 – 0.788)	<0.001	<0.01	2	
VEGFA rs833061	OR	0.834 (0.324 – 2.149)	0.707	76.02	2	
RAD51 rs1801320	OR	0.656 (0.349 – 1.232)	0.189	41.43	3	
FAS/APO-1 rs2234767	OR	1.001 (0.956 – 1.068)	0.982	0.00	3	
MMP-12 rs2276109	OR	1.588 (0.694 – 3.630)	0.273	88.80	2	
VEGF rs3025039	OR	0.869 (0.719 – 1.04)	0.144	0.00	2	
VDR rs7975232	OR	0.990 (0.901 – 1.088)	0.842	0.00	5	
VDR rs11568820	OR	1.164 (1.087 – 1.248)	<0.001	0.00	4	
XRCC2r rs3218536	OR	0.887 (0.750 – 1.050)	0.163	51.57	3	
*Other factors*						
Acrylamide	RR	0.994 (0.930 – 1.063)	0.865	0.00	2	
Obesity	RR	1.274 (1.194 – 1.36)	<0.001	0.00	2	
Overweight	OR	1.079 (1.041 – 1.119)	<0.001	24.04	3	
	RR	1.071 (1.041 – 1.102)	<0.001	0.00	3	
Height	RR	1.128 (1.064 – 1.196)	<0.001	87.71	3	
Weight	RR	1.067 (0.977 – 1.165)	0.149	74.99	2	
Smoking	RR	1.311 (0.847 – 2.029)	0.225	98.13	3	
Recreational physical activity	RR	0.830 (0.745 – 0.925)	<0.001	0.00	3	
Perineal talc	OR	1.297 (1.242 – 1.355)	<0.001	0.00	2	
	RR	1.250)1.177 – 1.327)	<0.001	38.11	2	
Breast feeding	OR	0.719 (0.679 – 0.762)	<0.001	4.63	4	

The risk of bias was assessed using the SIGN checklist. Among 277 included studies, 24.19 %, 39.35 %, and 36.46 % had “low quality”, “acceptable” and “high quality,“ respectively.

## Discussion

This study focuses on OC risk factors and protective measures. The factors can be classified into nutritional, drug use and medical history, diseases, and genetic. As regards nutritional factors, intake of coffee, egg, and fat can significantly enhance the risk of OC. Estrogen and estrogen-progesterone therapies (generally, hormone therapy) are also associated with the elevated risk of OC. Several diseases (e.g., diabetes, endometriosis, and polycystic ovarian syndrome), as well as some genetic polymorphisms (e.g., BRCA2 N372H rs144848, BSML rs1544410, Fokl rs2228570, MTHFR C677T, P16INK4a, ERCC2 rs13181, MMP-12 rs2276109, and VDR rs11568820), can significantly increase the incidence of OC. Other factors, like obesity, overweight, smoking, and the use of perineal talc, are also accompanied by an increased risk of OC.

Coffee is rich in several anti-oxidant and anti-carcinogenic bioactive compounds (e.g., phenolic acids, cafestol, and kahweol, respectively) [6]. This beverage has shown an inverse correlation with liver and endometrial cancer risk [4]. Furthermore, coffee and caffeine have an inverse relationship with sex hormones (testosterone and estradiol) [2]. High levels of these hormones have exhibited direct association with enhanced breast and ovarian cancer [8, 9]. Coffee contains acrylamide, which has been shown to increase the risk of breast and ovarian cancer as well [10]. The meta-analysis in the present study indicates a positive correlation between coffee drinking and OC risk.

Eggs are rich in cholesterol and choline, thus providing quite high protein per energy content, all of which are linked to the risk of breast, ovarian, and prostate cancers. Nonetheless, the majority of these studies on the mentioned cancers have not explored egg consumption as a primary exposure of interest, restricting a robust assessment of the hypothesized correlations. Since eggs have been considered as a source of protein and fat, its intake association with the OC risk has been primarily explored to examine the impact of protein or fat [11]. In this meta-analysis, egg consumption has been shown to be significantly and positively correlated with OC.

As one of the most controversial nutritional factors, dietary fat can enhance the development of hormone-related cancers (e.g., breast, endometrial, and OCs). However, the reports on this field are discrepant. High-fat diets may stimulate over-secretion of ovarian estrogen, leading to tumor-promoting mechanisms through mitogenic impacts on ERα- positive or negative tumor cells [12].

Epidemiologic reports indicate an association between estrogen exposure duration and OC induction and biology [13]. Recent research has expressed that besides inhibiting estrogen-driven growth in the uterus, progesterone can protect the ovaries against neoplastic transformation [14]. Despite the available poor knowledge of the etiology of OC, the role of estrogen and progestin seems biologically plausible. Based on a theory, high levels of menopausal gonadotropins due to estradiol expression may elevate OC risk. In other words, HRT can decrease the risk of OC by reducing the levels of menopausal gonadotropins. However, due to small HRT-related decrease, the mentioned advantages could be overruled by the estrogen-induced proliferation of ovarian cells. Moreover, the epithelial surface of both normal and malignant ovaries expresses estrogen receptors [15]. Furthermore, progestin is responsible for the declined risk associated with oral contraceptive use. Pregnancy can also offer a biologic basis for weak correlations with HRT formulations, including progestins [16]. The current work indicates a significant positive association between hormone therapy (estrogen, estrogen-progestin, and overall) and OC.

Diabetes mellitus (DM) is also positively and significantly associated with the risk of OC. Although the carcinogenic influence of DM on the ovary has not been completely understood, some mechanisms have been introduced to describe it partially. Hyperinsulinemia (often associated with insulin resistance) is commonly observed in type 2 DM patients. Chronic hyperinsulinemia has an association with tumor promotion due to the oncogenic potentials of insulin by stimulating cellular signaling cascade or incrementing growth factor-related cell proliferation [17]. Moreover, increased levels of insulin are associated with high bioactivity of insulin growth factor-1 (IGF-1) [18]. Considering the anti-apoptosis and mitogenic influences of IGF-1 on normal and cancerous human cells, type 2 DM can promote tumor development [19]. Besides, hyperglycemia has been recognized as one of the major health consequences of DM. Based on numerous animal and clinical studies, hyperglycemia is related to oxidative stress [20]. Oxidative stress refers to an imbalance between the reactive oxygen species (ROS) production and antioxidant defense mechanisms. ROS can damage the biomolecules of the cells, including those involved in cell proliferation and repair [21].

Based on the results, the risk of developing OC is 43 % in women with endometriosis. The endometriosis mechanisms in epithelial OC can be divided into 3 types. The first one is estrogen-dependent. Ness et al. introduce endometriosis as a precursor for epithelial OC, which is easily developed in the low-progesterone and high-estrogen conditions [22]. The second involves the genetic mutation in endometriotic tissues, like hepatocyte nuclear factor-1β (HNF-1β) [23] and *ARID1A* [24]. Furthermore, chronic inflammations, heme, or free iron-induced oxidative stress in endometriotic tissues also exhibit an association with epithelial OC [25].

The risk of OC shows a 60 % increase in women suffering from polycystic ovary syndrome (PCOS). PCOS has various risk factors, including obesity, diabetes, inflammation, metabolic syndrome, and aging. However, it is not clear whether the elevated risk of endometrial cancer is due to separate risk factors (e.g., diabetes, obesity) or PCOS itself. PCOS has its own metabolic characteristics, including hyperinsulinism, hyperglycemia, insulin resistance, and hyperandrogenism, enhancing cancer risk. Moreover, such a relationship between PCOS and endometrial cancer could be due to common inherited genetic variants. Other factors, such as parity (nulliparous versus multi), age at first pregnancy, and use/length of hormone therapy (HRT, OCP), could confound the results.

Some genetic factors may enhance the risk of developing OC. In the present study, Asn680Ser, *BRCA2* N372H rs144848, *BSML* rs1544410, *Fokl* rs2228570, *GSTM1*, *MTHFR* C677T, *NFƙB1*, *P16*^*INK4a*^, *ERCC2* rs13181, *MMP-12* rs2276109, and *VDR* rs11568820 have been found to increase the risk of OC significantly. Among the mentioned polymorphisms, P16INK4a has the strongest impact on the risk of OC (2.6-fold increase), followed by *NFƙB1* and *MMP-12.* rs2276109.

Some studies have mentioned the crucial role of *p16*^*INK4a*^ inactivation as the result of aberrant hypermethylation in the lung, liver, stomach, breast, and uterus carcinogeneses [26, 27]. In a meta-analysis on 6 eligible research encompassing 261 patients, Hu et al. show a correlation between *p16*^*INK4a*^ promoter hypermethylation and elevated risk of endometrial carcinoma [27]. A meta-analysis by Xiao et al. also report the significant association of aberrant methylation of *p16*^*INK4a*^ promoter with OC [28]. This could be regarded as a potential molecular marker for monitoring the diseases and providing new insights into OC therapies.

NFκB1 can significantly inhibit cell apoptosis through regulation of the level of survival genes, such as BCL-2 homolog A1, PAI-2, and IAP family. Moreover, studies have indicated the role of the NFκB1 signaling pathway in cellular proliferation by IL-5 enhancement, MAPK phosphorylation, and cyclin D1 expression modulation [29].

Numerous meta-analyses have addressed the relationship between NFκB1 promoter -94ins/del ATTG polymorphism and cancer risk, although their findings are not entirely consistent. For instance, Yang et al. [30] and Duan et al. [31] express that the polymorphism in NFκB1 -94ins/del ATTG promoter can increase the overall cancer risk. These results do not agree with those reported by Zou et al. [32]. Such contradictions can be assigned to the bias as the result of a limited sample size.

MMP-12 is involved in the pro-tumorigenesis process through inhibiting cancer cell apoptosis and promoting cancer cell invasion and migration [33]. As SNP of MMP-12-82 A>G can influence the MMP-12 expression and enhance the cancer risk, the correlation between MMP-12 promoter gene polymorphism and the cancer risk has been extensively addressed in recent years.

Obesity, overweight, smoking, and the use of perineal talc could be mentioned as other factors associated with OC risk. The biological mechanisms underlying the relation of overweight and obesity with OC are not clarified and consistent. Based on a study by Kuper et al. [34], progesterone and leptin could be possible endocrine mediators of the weight effect on OC risk. Such an impact could be assigned to elevated insulin levels, androgens, and free IGF-I due to obesity [35]. Regarding disassociation of BMI with OC risk among postmenopausal women, Reeves et al. [36] express that association of BMI with OC risk is under the mediation of hormones, as its impact on OC risk remarkably differs in premenopausal and postmenopausal subjects. BMI shows an inverse association with sex hormone-binding globulin and progesterone, while it is positively correlated with free testosterone in premenopausal women [37]. The mentioned hormone factors seem to be independently or cooperatively involved in the carcinogenic process.

Concerning biological mechanisms, the direct correlation of smoking with mucinous tumors can be assigned to the similarity of this neoplasm with cervical adenocarcinoma and colorectal cancers [38], both of which have exhibited direct association with tobacco exposure. Similarly, endometriois and clear cell cancers have some biological similarities with endometrial cancer, which is inversely related to tobacco smoking due to the possible anti-estrogenic influence of smoking. The tobacco smoking could exert strong impacts in the early stages of (ovarian) carcinogenesis. Thus, the more powerful tobacco-associated risk for mucinous could be explained by the fact that for the mucinous histotype, there is a continuum from benign to borderline and invasive disease, while serous OCs are often high grade and not originated from the borderline tumors [39]. Furthermore, the smoking-induced mutation in the somatic *KRAS* gene is more common in mucinous rather than serous borderline ovarian tumors [40], and also in borderline tumors than invasive cancer [41].

The ovarian carcinogenesis mechanism of perineal talc use has remained unclear. Based on a hypothesis, however, as an external stimulus, talc can ascend from the vagina to the uterine tubes and trigger a chronic inflammatory response, further promoting the OC development. Cellular injuries, oxidative stresses, and local elevation of inflammatory mediators (e.g., cytokines and prostaglandins) could be mutagenic, thus encouraging carcinogenesis [42]. Supporting this hypothesis, hysterectomy or bilateral tubal ligation, which may dramatically decline the ovarian exposure to inflammatory mediators, is related to a decreased OC risk [43–45].

## Conclusions

Numerous studies have addressed the effective factors of OC; however, these works have resulted in contradicting outcomes. The current study explores all previous meta-analyses and systematic reviews to provide a valuable summary of the OC protective and risk factors, among which nutritional and genetic factors play a more profound role. Although the genetic factors cannot be changed due to their inheritance, nutritional ones could be well regulated to prevent OC.

## Supplementary Information

**Additional file 1.**

## Data Availability

The data for supporting the research findings are available from the corresponding author upon reasonable request.
