# Supplementary material for: Investigation on factors associated with ovarian cancer: an umbrella review of systematic review and meta-analyses
Source: J Ovarian Res. 2021 Nov 11;14:153. doi: 10.1186/s13048-021-00911-z (PMC8582179; doi:10.1186/s13048-021-00911-z)
Supplement: Supplementary file 1 — Additional file 1. [file 13048_2021_911_MOESM1_ESM.docx]

**Table 1S.** Search Strategy in Medline (PubMed‎)

| No | Search Strategy *(PubMed‎)* |
| --- | --- |
|  |  |
| **1** | Ovary [Title/Abstract] OR ovarian [Title/Abstract] ‎ |
| **2** | Cancer* [Title/Abstract] OR carcinom*[Title/Abstract] OR tumor*[Title/Abstract] OR ‎Neoplas*[Title/Abstract] OR Malignanc*[ Title/Abstract ]‎ |
| **3** | Overview[Title/Abstract] OR systematic[Title/Abstract] OR review[Title/Abstract] OR ( meta [Title/Abstract] AND analysis[Title/Abstract]) |
| **4** | Prognos*[Title/Abstract] OR epidemiolog*[Title/Abstract] OR etiolog*[Title/Abstract] OR prevalence[Title/Abstract] OR incidence[Title/Abstract] OR risk[Title/Abstract] OR factor*[Title/Abstract] OR probabilit*[Title/Abstract] OR determinant*[Title/Abstract] OR predict*‎[Title/Abstract] |
| **5** | 1 AND 2 AND 3 |

**Table 2S.** List of factors which had reported just one meta-analysis

| *NO.* | *Factors* | *Description* | *Study* | *Measure of association* | *ES* | *95% CI* | |
| --- | --- | --- | --- | --- | --- | --- | --- |
|  |  |  |  |  |  | ***LCI*** | ***UCI*** |
| 1 | Fish Intake | Cohort Studies | Pei-yue Jiang (2014) | RR | 1.04 | .89 | 1.22 |
|  |  | Case-Control Studies | Pei-yue Jiang (2014) | RR | .9 | .73 | 1.12 |
| 2 | Meat consumption | Red meat | A Wallin (2011) | OR | 1.02 | .99 | 1.04 |
|  |  | Processed meat | A Wallin (2011) | OR | 1.05 | .98 | 1.14 |
|  |  | Overall meat | A Wallin (2011) | OR | 1.02 | 1 | 1.05 |
| 3 | Circulating vitamin D | increase of circulating 25(OH)D by 20 ng/ml | Lu Yin (2011) | RR | .83 | .63 | 1.08 |
| 4 | dietary flavonoids |  | Xiaoli Hua (2016) | RR | .82 | .68 | .98 |
| 5 | Type 2 Diabetes |  | Dongyu Zhang (2017) | RR | 1.24 | 1.06 | 1.44 |
| 6 | Dietary calcium |  | Xingxing Song (2017) | RR | .78 | .69 | .88 |
| 7 | Dairy calcium |  | Xingxing Song (2017) | RR | .8 | .66 | .98 |
| 8 | Total calcium intake |  | Xingxing Song (2017) | RR | .9 | .65 | 1.24 |
| 9 | serum levels of osteopontin |  | Yue-Dong Wang (2014) | SMD | 2.6 | 1.88 | 3.32 |
| 10 | dietary lycopene |  | Xinli Li (2014) | OR | .963 | .859 | 1.08 |
| 11 | Phytoestrogen Intake |  | Xin-Lan Qu (2014) | RR | .7 | .56 | .87 |
| 12 | folate intake |  | Chenglin Li (2013) | RR | 1.04 | .87 | 1.23 |
| 13 | Waist-to-hip ratio |  | Dagfinn Aune (2015) | RR | 1 | .93 | 1.07 |
| 14 | Hip circumference |  | Dagfinn Aune (2015) | RR | 1.04 | .82 | 1.32 |
| 15 | Waist circumference |  | Dagfinn Aune (2015) | RR | 1.06 | 11 | 1.12 |
| 16 | Exposure to Asbestos |  | M. C. Camargo (2011) | SMR | 1.77 | 1.37 | 2.28 |
| 17 | Paracetamol use |  | Stefanos Bonovas (2005) | RR | .84 | .7 | 1 |
| 18 | Reproductive Technology |  | Sonya Kashyap (2004) | OR | 1.52 | 1.18 | 1.97 |
| 19 | family history | first degree relatives | John F. Stratton (1998) | RR | 3.1 | 2.6 | 3.7 |
| 20 | dietary protein intake |  | Yanyang Pang (2018) | OR | .915 | .821 | 1.021 |
| 21 | N parity |  | Ho Kyung Sung (2016) | OR | .72 | .65 | .79 |
| 22 | dietary inflammatory index |  | Dongyang Li (2018) | RR | 1.57 | 1.23 | 2 |
| 23 | Benzodiazepine use |  | Hong-Bae Kim (2017) | OR | 1.09 | .94 | 1.26 |
| 24 | salpingectomy |  | Sang-Hee Yoon (2016) | OR | .51 | .35 | .75 |
| 25 | in vitro fertilization (IVF) |  | Li Li Li (2013) | RR | 1.59 | 1.24 | 2.03 |
| 26 | Birth weight |  | T. O. Yang (2014) | RR | .97 | .84 | 1.12 |
| 27 | renal transplants |  | Yu Wang (2018) | SIR | 1.6 | 1.23 | 2.07 |
| 28 | bisphosphonates use |  | Xiao-san Zhang (2018) | RR | .81 | .58 | 1.14 |
| 29 | Blood Groups | A vs non-A | Bei-bei Zhang (2014) | OR | 1.16 | 1.04 | 1.27 |
|  |  | B vs non-B | Bei-bei Zhang (2014) | OR | 1.05 | .9 | 1.2 |
|  |  | O vs non-O | Bei-bei Zhang (2014) | OR | .97 | .81 | 1.13 |
|  |  | AB vs non-AB | Bei-bei Zhang (2014) | OR | .76 | .53 | 1 |
| 30 | BMI (5 unit) |  | Dagfinn Aune (2015) | RR | 1.07 | 1.03 | 1.11 |
| 31 | isoflavones |  | Xin-Lan Qu (2014) | RR | .63 | .46 | .86 |
| 32 | lactose |  | L-Q Qin (2005) | RR | .94 | .72 | 1.24 |
| 33 | galactose |  | L-Q Qin (2005) | RR | .93 | .73 | 1.18 |
| 34 | antidepressant medication |  | Yun-Long Huo (2018) | OR | 1.1 | .9 | 1.32 |
| 35 | Hair Dyes |  | Bahi Takkouche (2005) | OR | .74 | .51 | 1.07 |
| 36 | hairdressers and related occupations |  | Bahi Takkouche (2009) | RR | 1.2 | 1.08 | 1.33 |
| 37 | Age at last birth |  | Yanjun Wu (2019) | RR | .77 | .65 | .91 |
| 38 | Intrauterine Device |  | Lindsay J. Wheeler (2019) | OR | .68 | .62 | .75 |
| 39 | vitamin C |  | Yuhang Long (2019) | RR | .95 | .81 | 1.11 |
| 40 | Work stress |  | Tingting Yang (2019) | RR | 1.05 | .72 | 1.53 |
| 41 | vitamin E |  | Youxu Leng (2019) | RR | .99 | .77 | 1.27 |
| 42 | fermented dairy food |  | Kui Zhang (2019) | OR | 1.01 | .83 | 1.22 |
| 43 | ESR2 rs1271572 |  | liang Tang (20118) | OR | 1.01 | .93 | 1.09 |
| 44 | HER2 rs1801200 (V655I) |  | liang Tang (20118) | OR | 1.15 | .64 | 2.07 |
| 45 | rs2279744 (SNP309) |  | Ying-Yu Ma (2013) | OR | .927 | .77 | 1.116 |
| 46 | MDM2 SNP285 (rs117039649) |  | Ping Wang (2016) | OR | .76 | .63 | .93 |
| 47 | rs1570360 |  | Chao-Huan Xu (2017) | OR | .83 | .22 | 3.22 |
| 48 | rs1799750 |  | Xu-Ming Zhu (2017) | OR | 1.02 | .83 | 1.25 |
| 49 | rs34093618 |  | Xu-Ming Zhu (2017) | OR | .97 | .68 | 1.38 |
| 50 | Androgen receptor gene CAG repeat polymorphism |  | Yang Deng (2017) | OR | .91 | .72 | 1.15 |
| 51 | BRCA1 P871L |  | Limin Miao (2017) | OR | 1.04 | .92 | 1.18 |
| 52 | p53 Codon 72 Arg.Pro Polymorphism |  | M. A. Alqumber (2014) | OR | 1.089 | .706 | 1.681 |
| 53 | TERT Genetic Polymorphism rs2853676 |  | Jin-Lin Cao (2015) | OR | 1.1 | 1.01 | 1.18 |
| 54 | XRCC3 Thr241Met (XRCC3-18067C/T) |  | Yulan Yan (2013) | OR | .98 | .94 | 1.03 |
| 55 | ERCC1 rs3212986 (C/A) |  | Fan Yang (2019) | OR | 1.25 | .93 | 1.69 |
| 56 | ERCC1 rs2298881 (A/C) |  | Fan Yang (2019) | OR | .72 | .37 | 1.38 |
| 57 | APC (adenomatous polyposis coli) promoter hypermethylation |  | Chunyan Shen (2016) | OR | 6.18 | 4.02 | 9.51 |
| 58 | IL-6 |  | Fangfang Zeng (2016) | RR | 1.16 | .89 | 1.5 |
| 59 | TNF-α |  | Fangfang Zeng (2016) | RR | 1.53 | .88 | 2.68 |
| 60 | TNFR2 |  | Fangfang Zeng (2016) | RR | 1.35 | .88 | 2.07 |
| 61 | CDH1 promoter methylation |  | Qiang Wang (2016) | OR | 8.71 | 4.87 | 15.58 |
| 62 | rs10895068 |  | Jing Liao (2014) | OR | 1.028 | .81 | 1.304 |
| 63 | Alu insertion |  | Jing Liao (2014) | OR | 1.504 | 1.026 | 2.203 |
| 64 | Val660Leu |  | Jing Liao (2014) | OR | 1.312 | .872 | 1.978 |
| 65 | rs2273535 |  | Jun Qin (2014) | OR | 1.08 | .96 | 1.22 |
| 66 | rs1047972 |  | Jun Qin (2014) | OR | .98 | .85 | 1.13 |
| 67 | COMT rs4680 |  | Jin-Ze Du (2014) | OR | .9 | .77 | 1.06 |
| 68 | Leu432Val |  | Ketan Gajjar (2012) | OR | .99 | .84 | 1.2 |
| 69 | Arg48Gly |  | Ketan Gajjar (2012) | OR | 1.1 | .9 | 1.3 |
| 70 | Ala119Ser |  | Ketan Gajjar (2012) | OR | 1.1 | .9 | 1.3 |
| 71 | Asn453Ser |  | Ketan Gajjar (2012) | OR | 1.2 | .92 | 1.6 |
| 72 | CYP1A1 MspI |  | T.N. Sergentanis (2012) | OR | 1.09 | .9 | 1.31 |
| 73 | CYP1A1 Ile462Val |  | T.N. Sergentanis (2012) | OR | 1.07 | .65 | 1.76 |
| 74 | CYP1A1 Thr461Asn |  | T.N. Sergentanis (2012) | OR | 1.52 | .92 | 2.53 |
| 75 | TP53 Arg72Pro |  | Su-Qin Shen (2012) | OR | 1.04 | .87 | 1.26 |
| 76 | GSTT1 |  | K. P. Economopoulos (2010) | OR | .93 | .8 | 1.09 |
| 77 | B7-H4 |  | Yun Ye (2018) | OR | 4.2 | 2.85 | 6.18 |
| 78 | CYP1A2*1F, rs762551 C/A |  | Zhong Tian (2013) | OR | .98 | .87 | 1.11 |
| 79 | klotho protein expression |  | Song Mao (2018) | OR | .616 | .434 | .874 |
| 80 | MMP3 -1171 5A>6A |  | Bo Peng (2010) | OR | .9 | .56 | 1.44 |
| 81 | MMP1 -1607 1G>2G |  | Bo Peng (2010) | OR | 1.21 | .86 | 1.71 |
| 82 | Prohibitin 3’ Untranslated Region C > T | not dominant model (TT vs (TC+CC)) | Tian-Biao Zhou(2012) | OR | 1.11 | .68 | 1.8 |
| 83 | rs1799768 |  | XIN XU (2012) | OR | .96 | .77 | 1.2 |
| 84 | PAI-1 4G/5G |  | Shangqian Wang (2013) | OR | 1 | .81 | 1.23 |
| 85 | IL-10 - 819C/T (rs1800871) |  | Zhibin Yu (2013) | OR | 1.47 | 1.02 | 2.1 |
| 86 | RAD51 (G172T) |  | Mengmeng Zhao (2014) | OR | 1 | .861 | 1.163 |
| 87 | Fas -670A.G (rs1800682) |  | Yeqiong Xu (2014) | OR | .87 | .66 | 1.15 |
| 88 | PON1-L55M |  | Lei Chen (2016) | OR | .913 | .55 | 1.517 |
| 89 | XRCC2 Arg188His |  | Yazhou He (2014) | OR | .83 | .73 | .95 |
| 90 | IL-27 (rs153109) A>G |  | Yun-Feng Zhang (2015) | OR | 1.04 | .74 | 1.48 |
| 91 | H19 polymorphisms (rs2107425, rs2839698 and rs217727) |  | Minjie Chu (2016) | OR | .84 | .68 | 1.05 |
| 92 | rs25487 (Arg399Gln) |  | Na-Na Yang (2017) | OR | .8 | .51 | 1.25 |
| 93 | rs1799782 (Arg194Trp) |  | Na-Na Yang (2017) | OR | .97 | .67 | 1.4 |
| 94 | rs25489 (Arg280His) |  | Na-Na Yang (2017) | OR | .77 | .62 | .95 |
| 95 | glutathione S-transferase P1 (GSTP1) Ile105Val |  | Erjiang Zhao (2017) | OR | 1.45 | .48 | 4.37 |
| 96 | MGMT promoter |  | Ru Chen (2017) | OR | 3.7 | 2.04 | 6.71 |
| 97 | transforming growth factor |  | Li-Ling Liu (2014) | OR | 2.3 | 1.01 | 5.22 |
| 98 | progesterone receptor (PGR) PROGINS |  | Ting Liu (2013) | OR | 1.09 | .97 | 1.19 |
| 99 | progesterone receptor (PGR) +331G/A |  | Ting Liu (2013) | OR | .99 | .74 | 1.32 |
| 100 | circulating insulin‑ growth factor 1 |  | QIAO WANG (2015) | OR | .85 | .51 | 1.4 |
| 101 | circulating insulin‑ growth factor binding protein 3 |  | QIAO WANG (2015) | OR | .78 | .43 | 1.4 |
| 102 | glutathione S-transferase M 1 null genotype |  | Yihua Yin (2013) | OR | 1.03 | .92 | 1.14 |
| 103 | MDM2 40-bp indel polymorphism |  | A. Moazeni-Roodi (2019) | OR | .91 | .77 | 1.09 |
| 104 | circulating omentin |  | M. Arjmand (2020) | SMD | -.02 | -.78 | .75 |
| 105 | mir-196a-2 rs11614913 |  | Jalal Choupani (2019) | OR | .95 | .34 | 2.63 |
| 106 | TERT rs10069690 |  | Guisheng He (2019) | OR | 1.14 | 1.1 | 1.19 |
| 107 | secreted frizzled‑related proteins (SFRPs) | SFRP1 | Jun Yu (2019) | OR | 59.7 | 23.59 | 151.07 |
|  |  | SFRP2 | Jun Yu (2019) | OR | 22.19 | 10.54 | 46.72 |
|  |  | SFRP4 | Jun Yu (2019) | OR | 19 | 6.54 | 55.16 |
|  |  | SFRP5 | Jun Yu (2019) | OR | 22.01 | 10.49 | 46.19 |
| 108 | Pelvic inflammatory disease |  | Zhiyi Zhou (2017) | RR | 1.24 | 1.06 | 1.44 |
| 109 | Night work |  | Lanhua Tang (2017) | RR | 1.24 | 1.08 | 1.43 |

**Table 3S.** List of factors which reported just in systematic reviews

| ID | Name of Factors | No. of articles | ID | Name of Factors | No. of articles |
| --- | --- | --- | --- | --- | --- |
| 1 | IGF-binding proteins [IGFBPs] | 1 | 28 | Sunlight | 1 |
| 2 | IL-18 -137G>C | 1 | 29 | Vitamin D | 2 |
| 3 | insulin growth factor [IGF] 1 | 1 | 30 | dietary risk factors | 1 |
| 4 | Lesbian and bisexual | 1 | 31 | Dietary glycaemic index | 1 |
| 5 | LIG4 gene | 1 | 32 | Green tea | 1 |
| 6 | mir-149 rs2292832 | 1 | 33 | Sweetened carbonated beverage | 1 |
| 7 | MLH1 -93 G>A | 1 | 34 | Dietary alkylresorcinols | 1 |
| 8 | NME1 | 1 | 35 | salpingectomy | 1 |
| 9 | Non-isoflavone flavonoids | 1 | 36 | serum hormones measured in pregnancy | 1 |
| 10 | Long noncoding RNA HAGLROS | 1 | 37 | HIV | 1 |
| 11 | Opportunistic Bilateral Salpingectomy | 1 | 38 | fertility drugs | 2 |
| 12 | Aurora-A V57I (rs1047972) | 1 | 39 | Cardiac glycosides | 1 |
| 13 | ovulation stimulating drugs | 1 | 40 | Cell cycle genes | 1 |
| 14 | predicted pathogenic PALB2 | 1 | 41 | DNA repair genes | 1 |
| 15 | Quercetin | 1 | 42 | EPHX1 polymorphism | 1 |
| 16 | rs11225395 | 1 | 43 | flavonoid and lignan | 1 |
| 17 | rs12278250 | 1 | 44 | glycaemic load | 1 |
| 18 | rs16917496 | 1 | 45 | vitamin D receptor | 1 |
| 19 | rs17860523 | 1 | 46 | H19 rs2107425 C>T | 1 |
| 20 | rs2292730 | 1 | 47 | assisted reproductive technologies (ART) | 1 |
| 21 | rs6094237 | 1 | 48 | HOX transcript antisense RNA (HOTAIR polymorphisms) | 1 |
| 22 | rs9787933 | 1 | 49 | Hypodontia | 1 |
| 23 | BHMT rs3733890 | 1 | 50 | Sex steroid hormone genes | 1 |
| 24 | bone morphogenetic protein-2 (rhBMP-2) | 1 | 51 | C-1562T | 1 |
| 25 | BRCA | 3 | 52 | C-735T | 1 |
| 26 | C-1306T | 1 | 53 | ARLTS1 polymorphism | 1 |
| 27 | TCF7L2 | 1 |  |  |  |
